# Dietary Supplementation With Citrus Extract Altered the Intestinal Microbiota and Microbial Metabolite Profiles and Enhanced the Mucosal Immune Homeostasis in Yellow-Feathered Broilers

**DOI:** 10.3389/fmicb.2019.02662

**Published:** 2019-11-26

**Authors:** Miao Yu, Zhenming Li, Weidong Chen, Gang Wang, Yiyan Cui, Xianyong Ma

**Affiliations:** ^1^Institute of Animal Science, Guangdong Academy of Agricultural Sciences, Guangzhou, China; ^2^State Key Laboratory of Livestock and Poultry Breeding, Guangzhou, China; ^3^Key Laboratory of Animal Nutrition and Feed Science in South China, Ministry of Agriculture, Guangzhou, China; ^4^Guangdong Key Laboratory of Animal Breeding and Nutrition, Guangzhou, China; ^5^Guangdong Engineering Technology Research Center of Animal Meat Quality and Safety Control and Evaluation, Guangzhou, China

**Keywords:** citrus extract, immune homeostasis, intestinal microbial community, microbial metabolites, yellow-feathered broilers

## Abstract

The present study aimed to investigate the effects of citrus extract (CE) on intestinal microbiota, microbial metabolite profiles, and the mucosal immune status in broilers. A total of 540 one-day-old yellow-feathered broilers were randomly allotted into three groups and fed a basal diet (control group), or a basal diet containing 10 mg/kg of zinc bacitracin (antibiotic group), or 10 mg/kg of CE (CE group). Each treatment consisted of six replicates, with 30 broilers per replicate. After 63-day feeding, two broilers per replicate were randomly selected and slaughtered, and their ileal and cecal digesta and ileal tissue were collected for microbial composition, microbial metabolites, and gene expression analysis. The results showed that CE significantly increased the abundance of *Barnesiella* and *Blautia* than did the antibiotic group (adjusted *P* < 0.05), whereas it decreased the abundance of *Alistipes* and *Bacteroides* (adjusted *P* < 0.05). Meanwhile, the CE group also increased the numbers of *Bifidobacterium* and *Lactobacillus* than did the control and antibiotic groups (*P* < 0.05), whereas it decreased the number of *Escherichia coli* (*P* < 0.05). For microbial metabolites, dietary supplementation with CE increased the concentrations of lactate, total short-chain fatty acids, acetate, and butyrate in the cecum than did the control and antibiotic groups (*P* < 0.05), whereas it decreased the concentrations of amino acid fermentation products (ammonia, amines, *p*-cresol, and indole) (*P* < 0.05). Additionally, supplementation with CE up-regulated (*P* < 0.05) the mRNA expression of intestinal barrier genes (*ZO-1* and *Claudin*) in the ileum than did both the control and antibiotic groups. However, antibiotic treatment induced gut microbiota dysbiosis, altered the microbial metabolism, and disturbed the innate immune homeostasis. In summary, these results provide evidence that dietary supplementation with CE can improve the intestinal barrier function by changing microbial composition and metabolites, likely toward a host-friendly gut environment. This suggests that CE may possibly act as an efficient antibiotic alternative for yellow-feathered broiler production.

## Introduction

In-feed antibiotics have been extensively used as growth promoters in livestock production to maintain health and to improve feed conversion efficiency, utilization, and growth performance (Castanon, 2007). However, the continuous and excessive use of in-feed antibiotics for animals’ production has led to the development of antibiotic-resistant microbes and a number of residual antibiotics in animal products, both of which pose a potential threat to human health (Gadde et al., 2017). In China, the use of antibiotics in poultry feeds is still a common practice, although it has increasingly caused safety concerns and increased the consumer demand for antibiotic-free animal products; thus, the use of antibiotics for growth promotion in feed will be banned in the future. Consequently, it is necessary to develop novel feed alternatives that offer both security and efficiency with the potential to replace antibiotics while improving poultry health and product quality.

As a safe and efficient alternative to antibiotics, many plant extracts have been used as promising feed additives for livestock production. Among these potential alternatives, citrus extract (CE) is often used as one of promising candidates in poultry (Akbarian et al., 2013; Ebrahimi et al., 2015; Pourhossein et al., 2015). CE is a rich source of many important bioactive ingredients including vitamins, minerals, phenolic compounds, nobiletin, and flavonoids (Li et al., 2006; Parmar and Kar, 2010; Bermejo et al., 2011). Thus, CE may provide numerous health benefits, including as an antimicrobial agent against *Escherichia coli* and *Salmonella typhimurium* with the ability to selectively inhibit the growth of potentially pathogenic bacteria (Nannapaneni et al., 2008, 2009; O’Bryan et al., 2008) and enhance immune system activities (Chen et al., 2012). Indeed, the beneficial effects of CE were extensively investigated in poultry production. Several previous studies reported that the dietary supplementation with citrus products in broiler feed could enhance growth performance (Seidavi et al., 2015), stimulate IgG and IgM antibody production in serum (Pourhossein et al., 2015), and decrease the number of *E. coli* in the cecum digesta by using a culture-based approach (Ebrahimi et al., 2015; Alefzadeh et al., 2016). These results indicated that CE can modulate the intestinal microbiota and immune system activities. However, the effects of CE on the intestinal microbial community and epithelial immune status remain limited and require further investigation. Additionally, alterations in the microbiota by dietary treatment can also induce changes in the metabolic end-products of microbial degradation (Fouhse et al., 2015). However, whether dietary supplementation with CE affects the intestinal microbial metabolites in broilers remains unclear.

To test the hypothesis that CE as an antibiotic alternative may positively alter the microbial community and its metabolites, and that these alterations can also modulate the mucosa immune response in yellow-feathered broilers, the current study investigated the effects of dietary supplementation with CE on the microbial community, microbial metabolite profiles, and expression of immune-related genes in the intestine.

## Materials and Methods

### Ethics Approval and Consent to Participate

The experimental proposals and procedures for the care and treatment of the broilers were approved by the Animal Care and Use Committee of Guangdong Academy of Agricultural Sciences (authorization number GAASIAS-2016-017).

### Animals, Experimental Design, and Sampling

A total of 540 one-day-old yellow-feathered male broilers were randomly allotted into three groups. Each treatment consisted of six replicates, and each replicate had 30 broilers. There was no difference of statistics in initial average body weight of broilers among the three group (41.37 ± 0.35 g). The broilers of the control group were fed a basal diet without any antibiotics (control group); the antibiotic and CE groups were fed the same basal diet with 10 mg/kg of zinc bacitracin (antibiotic group) and 10 mg/kg of CE (CE group) during the whole trial period, respectively. The CE used in the current study was provided by the Guangdong Runsen Health and Environmental Technology Development Co., Ltd., Guangdong, China. The contents of total flavone, polysaccharide, citric acid, and chlorogenic acid in the CE were measured as previously described (Kong et al., 2009; Wan et al., 2016) and were 2.48, 1.20, 1.30, and 0.68%, respectively. The basal diets were formulated to either meet or exceed the nutrient requirements of Chinese yellow-feathered broilers (Ministry of Agriculture of P. R. China, 2004). The dietary composition and nutrient contents for the starting (1–21 days), growing (22–42 days), and finishing (43–63 days) phases are shown in Table 1. All broilers were housed in battery cages (3.0 m × 3.0 m × 0.9 m) in an environmentally controlled room with a continuous light regimen throughout the 63-day experimental period. The environment temperature was maintained at 33°C for the first week and then decreased by 3°C every week until a final temperature of 24°C. All broilers were provided with diets and water *ad libitum* throughout the whole trial period.

**TABLE 1:** Feed ingredient and nutrient levels of experimental diets used in different phases of trial (%, as-fed basis).

**Items**	**1–21 days**	**22–42 days**	**43–63 days**
**Ingredients**			
Corn	60.00	65.00	69.20
Soybean meal	29.00	23.40	18.50
Fish meal	1.80	–	–
Corn gluten meal	2.00	4.00	4.00
Soybean oil	1.36	3.00	2.82
L-Lysine HCl (98%)	0.07	0.18	0.15
DL-Methionine	0.20	0.09	0.13
Salt	0.28	0.27	0.27
Dicalcium phosphate	1.68	1.74	1.60
Limestone	1.18	1.12	1.11
Zeolite	1.43	0.20	1.22
Vitamin-mineral premix^1^	1.00	1.00	1.00
Total	100.00	100.00	100.00
**Calculated composition^2^, %**			
ME/(MJ/kg)	12.13	12.55	12.97
Lysine	1.10	0.95	0.85
Methionine	0.55	0.48	0.40
Ca	1.00	0.90	0.81
TP	0.68	0.64	0.57
AP	0.45	0.40	0.34
**Analyzed composition, %**			
CP	20.96	19.21	17.49

*ME, metabolized energy; TP, total phosphorus; AP, available phosphate. ^1^Provided per kilogram of complete diet: pantothenic acid, 10.9 mg; nicotinic acid, 30 mg; folic acid, 0.95 mg; biotin, 0.16 mg; vitamin A, 8,000 IU; vitamin D, 2,800 IU; vitamin E, 19 mg; vitamin K3, 3.32 mg; vitamin B1, 1.7 mg; vitamin B2, 8.2 mg; vitamin B6, 2.78 mg; vitamin B12, 0.015 mg; Mn (MnSO_4_⋅H_2_O), 82 mg; Zn (ZnSO_4_⋅H_2_O e), 68 mg; Fe (FeSO_4_⋅H_2_O), 81 mg; Cu (CuSO_4_⋅5H_2_O), 9 mg; I (KI), 0.50 mg; Se (Na_2_SeO_3_), 0.27 mg. ^2^Values were calculated from data provided by Feed Database in China (2016) except that crude protein was analyzed.*

At the end of the feeding period (day 63), two broilers per replicate were randomly selected and slaughtered after being fasted for approximately 12 h. The ileal and cecal digesta were collected, homogenized, and stored at –80°C for later determination of the microbial communities and metabolites analyses. In addition, a segment of mid-ileum tissue was also rapidly removed and washed with phosphate-buffered saline (PBS, pH 7.0), then immediately frozen in liquid nitrogen, and stored at –80°C for later gene expression.

### Intestinal Microbial DNA Extraction, Illumina MiSeq Sequencing, and Bioinformatics Analysis

The microbial total genomic DNA extraction was conducted from 250 mg of ileal and cecal digesta samples, using the QIAamp PowerFecal DNA Kit (Qiagen, Hilden, Germany), according to the manufacturer’s instructions. The concentrations of every DNA sample were quantified using a NanoDrop 2000 spectrophotometer (Thermo Scientific, Wilmington, DE, United States). To balance the cost of the experiment and the number of replicates necessary, two samples from each replicate were pooled in equal proportion and were selected for 16S rRNA MiSeq sequencing. The V3–V4 region of the bacterial 16S rRNA gene was amplified using primer pairs 338F (5′-ACTCCTRCGGGAGGCAGCAG-3′) and 806R (5′-GGACTACCVGGGTATCTA AT-3′) (Mao et al., 2015). PCR products were purified using an AxyPrep DNA Gel Extraction Kit (Axygen Biosciences, Union City, CA, United States) to remove excess primer dimers and dNTPs. After purification, the amplicons were pooled in equimolar and performed on an Illumina MiSeq 2 × 250 platform (Illumina, San Diego, CA, United States) at Majorbio Bio-Pharm Technology (Shanghai, China) according to standard protocols (Caporaso et al., 2012). The raw reads in this study were submitted to the National Center of Biotechnology Information (NCBI) Sequence Read Archive (SRA) database under accession number SRR9074899–SRR9074916.

QIIME (version 1.17) software package was used to demultiplex and quality filter the obtained sequences from 18 samples according to a previous study (Mu et al., 2016). Chimeric sequences were identified and removed using UCHIME (Edgar, 2010), and the high-quality sequences were clustered into operational taxonomic units (OTUs) with a cutoff of 97% similarity using UPARSE (version 7.1)^1^. Each OTU was annotated using the Ribosomal Database Project (RDP) classifier against the Silva (SSU119) 16S rRNA database at a confidence threshold of 90%. The diversity of cecal microbiota (such as rarefaction analysis, ACE and Chao1 richness estimators, and Shannon and Simpson diversity indices) was performed using Mothur (version 1.36.1) according to a previous study (Schloss et al., 2009). Beta diversity was evaluated by principal coordinate analysis (PCoA) based on the Bray–Curtis distance. An unweighted distance-based analysis of molecular variance (AMOVA) was conducted to compare the significant differences between samples by using Mothur (Schloss et al., 2009). Linear discriminant analysis effect size (LEfSe) analysis was employed to explore the significantly different bacteria at the OTU level among the three groups (Segata et al., 2011).

### Quantification of Microbes by Real-Time PCR

To identify the quantitative changes in bacterial groups, several key bacteria groups, such as total bacteria, Firmicutes, Bacteroidetes, *Clostridium* cluster IV, *Clostridium* cluster XIVa, *E. coli*, *Bifidobacterium*, *Lactobacillus*, *Prevotella*, and *Ruminococcus*, in the ileal and cecal digesta were quantified by real-time quantitative PCR using specific primers (Supplementary Table S1). qPCR was performed by using TB Green^TM^ Premix Ex Taq^TM^ (Takara Biotechnology, Dalian, China) on the CFX96 Real-Time PCR Detection System (Bio-Rad, Hercules, CA, United States). The real-time PCR mixtures and conditions were set according to previously described methods (Yu et al., 2017b; Song et al., 2019a).

### Analysis of Cecal Microbial Metabolites

Cecal digesta samples were analyzed for microbial metabolites. Short-chain fatty acids (SCFAs) concentrations were measured by gas chromatography (GC) according to a method described previously (Yu et al., 2017b). Lactate concentration was determined using a commercial assay kit (Nanjing Jiancheng Biological Engineering Institute, Nanjing, China) according to the manufacturer’s instructions. Ammonia concentration was analyzed using a UV spectrophotometer (Shimadzu, Tokyo, Japan) according to the description of previous study (Chaney and Marbach, 1962). Biogenic amine concentrations were measured using high-performance liquid chromatography (HPLC) with precolumn dansylation method as previously described (Yang et al., 2014). The concentrations of phenolic and indolic compounds were determined by HPLC according to a method that has been previously described (Schüssler and Nitschke, 1999).

### RNA Extraction and qPCR for Ileal Gene Expression

The total RNA of ileal tissue was extracted using TRIzol reagent (Takara Biotechnology, Dalian, China) according to the method described by manufacturer’s instructions. The RNA concentration and purity were quantified using NanoDrop 2000 spectrophotometer (Thermo Scientific, Wilmington, DE, United States), and the absorption ratio (OD260:OD280) of all samples ranged from 1.8 to 2.0. One microgram total RNA was reverse-transcribed using the PrimeScript^TM^ RT reagent Kit with gDNA Eraser (Takara Biotechnology, Dalian, China). The specific primer sequences used in the current study are presented in Supplementary Table S2. The target genes and GAPDH were determined by quantitative real-time PCR with TB Green^TM^ Premix Ex Taq^TM^ (Takara Biotechnology, Dalian, China), and fluorescence was detected on CFX96 Real-Time PCR Detection System (Bio-Rad, Hercules, CA, United States). The reaction condition and real-time PCR condition were previously described (Yu et al., 2017a). GAPDH mRNA expression levels were used as a housekeeping gene. The results of target genes mRNA expression level calculated using the 2^(–ΔΔCt)^ method, where ΔΔCt = (Ct_*target*_ − Ct_*GAPDH*_)_*treatment*_ − (Ct_*target*_ − Ct_*GAPDH*_)_*control*_.

### Statistical Analysis

Experimental data were analyzed using the IBM SPSS statistics V20.0.0 software package (IBM Corp., Armonk, NY, United States). The Shapiro–Wilk test was used to assess whether all variables exhibited a normal distribution before assessing differences among the three groups. Then, the variables that showed a non-normal distribution were analyzed by Kruskal-Wallis one-way analysis of variance (ANOVA). To avoid type I errors, the resulting *P* values of bacterial abundance were adjusted via the Benjamini–Hochberg false discovery rate (FDR) multiple-testing correction (Benjamini and Hochberg, 1995). Bacterial abundance data are expressed as medians, and an FDR-adjusted *P* value < 0.05 was regarded as significant. The data of bacterial gene copy, microbial metabolites (SCFAs, lactate, and amino-acid derived metabolites), and ileal gene expression were analyzed via one-way ANOVA with a Tukey *post hoc* test. Differences were regarded significant at *P* < 0.05.

## Results

### Growth Performance

During the whole trial, the outward appearance of broilers was healthy, and no mortality was observed. In the current study, dietary supplementation with CE significantly increased (*P* < 0.05) the average daily gain (ADG) of broilers (means ± SEM: 32.12 ± 0.35 and 34.59 ± 0.71 g/day in the control and CE groups, respectively) and decreased (*P* < 0.05) the feed conversion rate (F:G, means ± SEM: 2.57 ± 0.05 and 2.39 ± 0.04 in the control and CE groups, respectively) than did the control group. The ADG and F:G ratio in the antibiotic group showed no difference (*P* > 0.05) than those in the control and CE groups. Furthermore, no difference (*P* > 0.05) in the average daily feed intake (ADFI) was observed among the control group (means ± SEM: 82.61 ± 1.11 g/day), antibiotic group (means ± SEM: 83.30 ± 1.67 g/day), and CE group (means ± SEM: 82.43 ± 1.56 g/day).

### Microbial Composition of Ileal and Cecal Digesta

**FIGURE 1:**
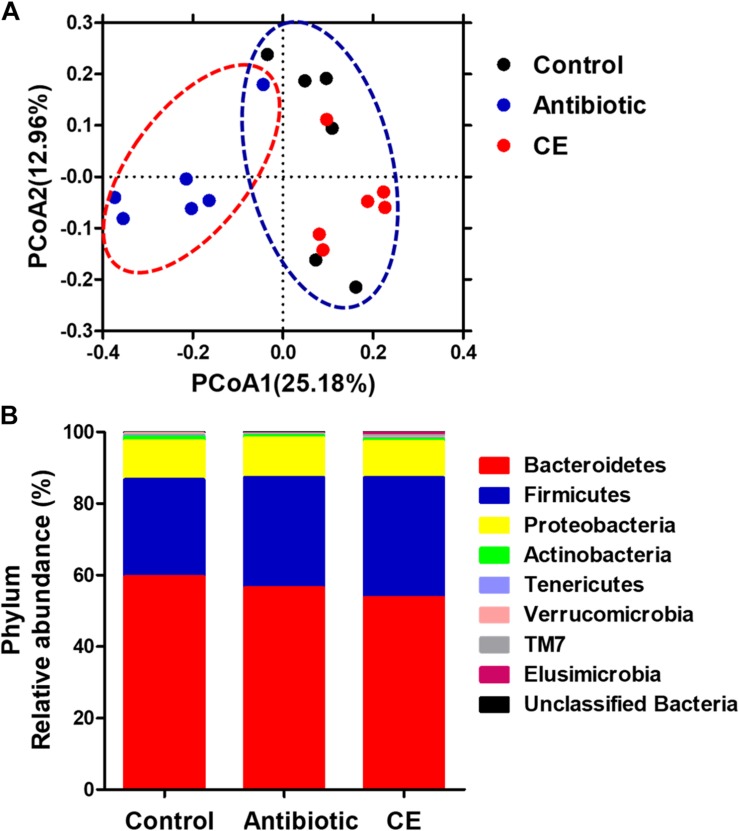
**(A)** Principal coordinates analysis (PCoA) of bacterial communities in the cecal digesta of Chinese yellow-feathered broilers (based on the Bray–Curtis distance). Circles with dashed or solid line indicate that groups were significantly distinct using AMOVA (*P* < 0.05). **(B)** Phylum-level relative abundance of 16S rRNA gene sequences from the cecal digesta of Chinese yellow-feathered broilers. CE, citrus extract; AMOVA, analysis of molecular variance.

To profile the microbial composition, the cecal microbiota of yellow-feathered broilers was analyzed by 16S rRNA MiSeq sequencing. In the current study, a total of 673,959 sequences from the 18 samples (with an average of 37,442 sequences per sample) after Illumina sequencing was revealed for the subsequent analyses. Mean-based rarefaction curves showed that the sampling of each group provided sufficient sequences to reflect the diversity and abundance of bacteria (Supplementary Figure S1). The ACE, Chao1 index, Shannon index, and Simpson index did not differ among the three different groups (Supplementary Figure S2). The PCoA with the Bray–Curtis distance indicated that the samples of antibiotic group gathered together and clearly separated from the samples of the control and CE groups (Figure 1A), suggesting that the microbial composition of yellow-feathered broilers in the antibiotic group differs from that of the control and CE groups. AMOVA also showed significant dissimilarities among the antibiotic, control, and CE groups (*F*s = 2.84, *F*s = 2.84, *P* < 0.001, among the control, antibiotic, and CE groups; *F*s = 4.07, *P* < 0.01, antibiotic vs. CE; *F*s = 3.07, *P* < 0.001, antibiotic vs. control; *F*s = 1.27, *P* > 0.05, control vs. CE).

At the phylum level (Figure 1B), eight phyla were identified: Bacteroidetes, Firmicutes, Proteobacteria, Actinobacteria, Tenericutes, Verrucomicrobia, TM7, and Elusimicrobia in the cecal digesta of yellow-feathered broilers. Among these phyla, Bacteroidetes, Firmicutes, and Proteobacteria formed the three dominant phyla, contributing 59.61, 27.11, and 10.84% in the control group; 56.46, 30.86, and 11.01% in the antibiotic group; and 53.78, 33.47, and 10.07% in the CE group, respectively. There were no significant changes in the abundances of any phyla among the three groups (*P* > 0.05).

**FIGURE 2:**
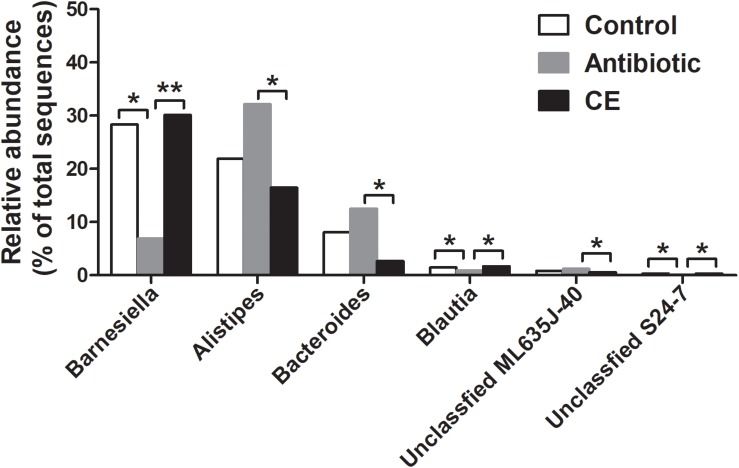
Significantly changed bacteria genera by citrus extract treatment. The values were expressed as the medians (*n* = 6). Asterisks indicate statistical differences between different groups (Kruskal–Wallis test): ^∗^FDR-adjusted *P* value < 0.05; ^∗∗^FDR-adjusted *P* value < 0.01. CE, citrus extract; FDR, false discovery rate.

At the genus level, the 30 most predominant genera of the cecal digesta are presented in a heat map (Supplementary Figure S3). The nine dominant genera (those with a relative abundance ≥ 2% in at least one group) included *Barnesiella*, *Alistipes*, *Staphylococcus*, *Bacteroides*, *Salmonella*, unclassified Ruminococcaceae, unclassified vadinBB60, unclassified Enterobacteriaceae, and unclassified Lachnospiraceae. Compared with that in the control group, the antibiotic treatment significantly decreased the abundance of *Barnesiella*, *Blautia*, and unclassified S24-7 in the cecum of broilers (adjusted *P* < 0.05) (Figure 2). Compared with that in the CE group, antibiotic treatment decreased the abundance of *Barnesiella*, *Blautia*, and unclassified S24-7 (adjusted *P* < 0.05), whereas it increased the abundance of *Alistipes*, *Bacteroides*, and unclassified ML635J-40 (adjusted *P* < 0.05).

**FIGURE 3:**
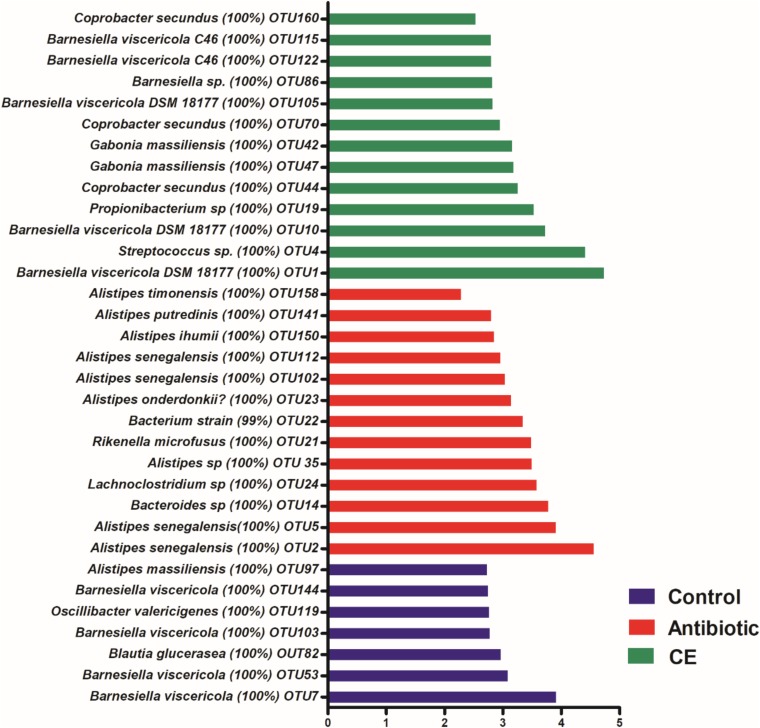
Significantly changed bacterial OTUs in the cecal digesta by citrus extract treatments, as revealed by LEfSe analysis. Only an LDA score of >2 was considered significant. CE, citrus extract; OTUs, operational taxonomic units; LDA, linear discriminant analysis; LEfSe, LDA effect size.

At the species level, 2,477 effective OTUs were identified in the cecal samples. To confirm specific bacteria that are characteristic for dietary treatment, LEfSe analysis was also performed at the species level (Figure 3). A total of 33 OTUs were different among the three groups. Among these different OTUs, seven OTUs were higher in the control group, 13 OTUs were higher in the antibiotic treatment group, and 13 OTUs were higher in the CE treatment group (adjusted *P* < 0.05).

**FIGURE 4:**
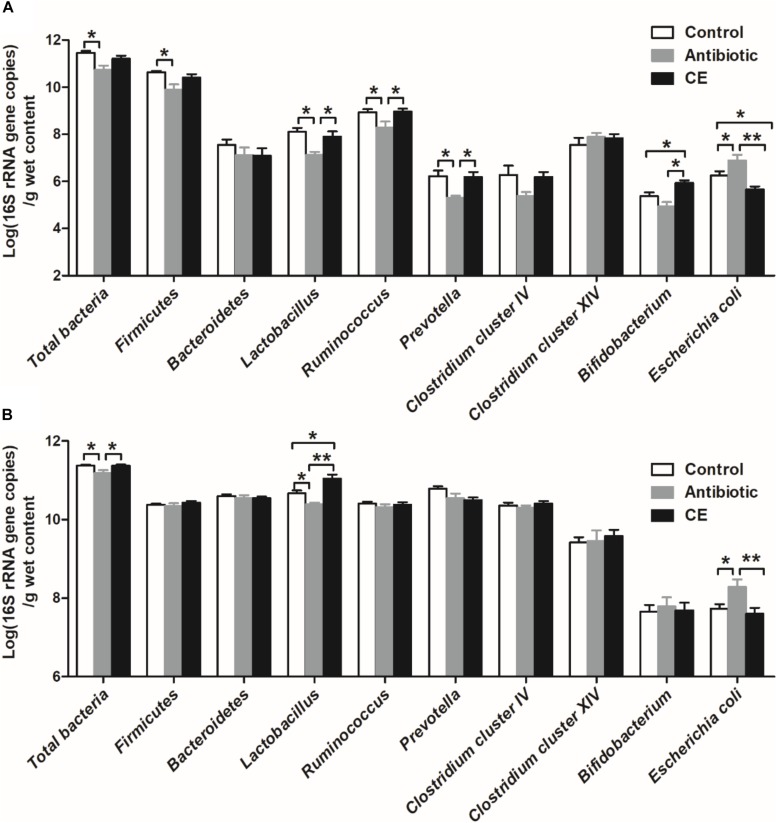
Response of the copy numbers (Log 10 gene copies/g digesta sample) of major bacterial taxonomic groups in the ileal **(A)** and cecal **(B)** digesta of Chinese yellow-feathered broilers toward citrus extract treatment. The values are means ± SEM (*n* = 6). Asterisks indicate statistical differences between different groups (one-way ANOVA with a Tukey *post hoc* test): ^∗^*P* < 0.05. CE, citrus extract.

Real-time PCR was used to identify the number changes of several key bacteria groups in the ileal and cecal digesta of broilers following treatment with antibiotic and CE. In the ileum (Figure 4A), antibiotic treatment significantly decreased the number of total bacteria, Firmicutes, *Lactobacillus*, *Ruminococcus*, and *Prevotella* compared with those in the control group (*P* < 0.05), whereas it increased the number of *E. coli* (*P* < 0.05). Antibiotic treatment also decreased the numbers of *Lactobacillus*, *Ruminococcus*, *Prevotella*, and *Bifidobacterium* (*P* < 0.05) compared with those in CE group, whereas it increased the number of *E. coli* (*P* < 0.05). Additionally, CE treatment increased the number of *Bifidobacterium* compared with those in control group (*P* < 0.05), whereas it decreased the number of *E. coli* (*P* < 0.05). The numbers of Bacteroidetes, *Clostridium* cluster IV, and *Clostridium* cluster XIV has no significant difference among the three groups (*P* > 0.05). Compared with the control and CE groups, antibiotic treatment significantly decreased the number of total bacteria and *Lactobacillus* in the cecum (Figure 4B) (*P* < 0.05), whereas it increased the number of *E. coli* (*P* < 0.05). Moreover, CE treatment increased the number of *Lactobacillus* compared with that in the control group (*P* < 0.05). However, there were no significant differences (*P* > 0.05) observed in the numbers of Firmicutes, Bacteroidetes, *Ruminococcus*, *Prevotella*, *Clostridium* cluster IV, *Clostridium* cluster XIV, and *Bifidobacterium* among the different dietary treatments. Overall, these results indicated that antibiotic and CE treatments significantly altered the intestinal bacterial community structure and the numbers of individual microbes.

### Microbial Metabolites in the Ileal and Cecal Digesta

**FIGURE 5:**
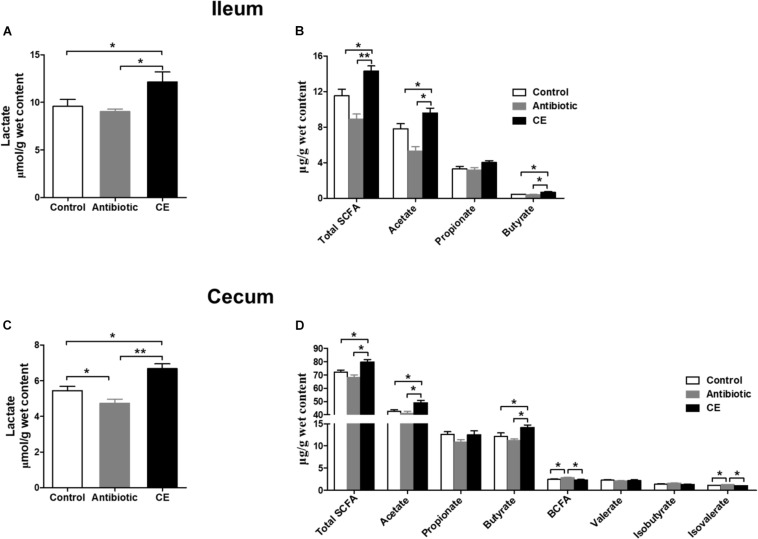
Effect of the dietary supplementation with citrus extract on the carbohydrate metabolites in the ileal and cecal digesta of Chinese yellow feathered broilers: **(A)** Ileal lactate, **(B)** Ileal SCFAs, **(C)** Cecal lactate, and **(D)** cecal SCFAs. The values are means ± SEM. Asterisks indicate statistical differences between different groups (one-way ANOVA with a Tukey *post hoc* test): ^∗^*P* < 0.05. Total SCFA, total short-chain fatty acid; BCFA, branched-chain fatty acid.

Lactate and SCFAs are the major carbohydrate fermentation products of gut microbes and serve as indicators of microbial activity. For lactate, dietary CE supplementation significantly increased the concentration of lactate compared with that in control and antibiotic groups in the ileum (Figure 5A) and cecum (Figure 5C, *P* < 0.05). Antibiotic treatment significantly decreased the lactate concentration in the cecum compared with that in the control group (*P* < 0.05). For SCFAs, dietary supplementation with CE significantly increased total SCFA, acetate, and butyrate concentrations in the ileum (Figure 5B) and cecum (Figure 5D) compared with those in control and antibiotic groups (*P* < 0.05), whereas it decreased branched-chain fatty acids (BCFAs) and isovalerate concentrations in the cecum compared with those in the antibiotic group (*P* < 0.05). Furthermore, antibiotic treatment increased the concentrations of BCFA and isovalerate compared with those in the control group (*P* < 0.05). However, the concentrations of propionate, valerate, and isobutyrate were not affected by different dietary treatments (*P* > 0.05).

**FIGURE 6:**
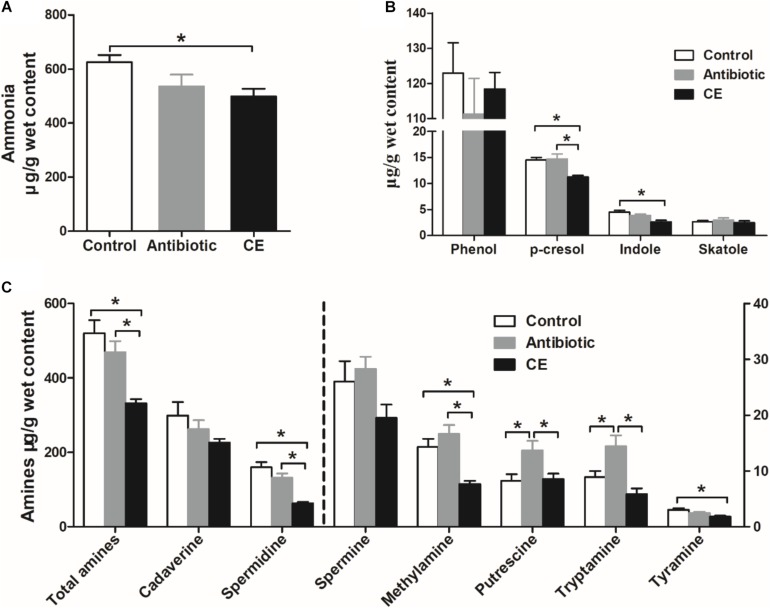
Effect of the dietary supplementation with citrus extract on the concentrations of amino acid fermentation products in the cecal digesta of Chinese yellow-feathered broilers: **(A)** ammonia, **(B)** phenolic and indole compounds, and **(C)** biogenic amines. The values are means ± SEM. Asterisks indicate statistical differences between different groups (one-way ANOVA with a Tukey *post hoc* test): ^∗^*P* < 0.05. CE, citrus extract.

Ammonia and biogenic amines are amino acid deamination and decarboxylation products of gut microbes, respectively, and phenolic and indolic compounds are a product of aromatic amino acid degradation by gut microbes. For ammonia (Figure 6A), dietary CE supplementation decreased the concentration of ammonia compared with that in the control group (*P* < 0.05), whereas no significant differences were observed between the control and antibiotic groups (*P* > 0.05). For phenolic and indolic compounds (Figure 6B), CE supplementation decreased the concentration of *p*-cresol compared with that in the control and antibiotic groups (*P* < 0.05) and decreased the concentration of indole compared with that in the control group (*P* < 0.05). Dietary treatment showed no effect on the concentrations of phenol and skatole (*P* > 0.05). For amines (Figure 6C), cadaverine and spermidine were the major amines in the cecal digesta of broilers. Dietary supplementation with CE significantly decreased total amines, spermidine, methylamine, and tyramine concentrations compared with those in the control group (*P* < 0.05) and decreased total amines, spermidine, methylamine, putrescine, and tryptamine concentrations compared with those in the antibiotic group (*P* < 0.05). Furthermore, antibiotic treatment increased putrescine and tryptamine concentrations compared with those in the control group (*P* < 0.05). However, the concentrations of cadaverine and spermine were not changed by different dietary treatments (*P* > 0.05). Overall, the results of the current study indicate that dietary supplementation with CE markedly enhanced the bacterial fermentation of carbohydrates and decreased the bacterial fermentation of protein in the cecum, whereas antibiotic treatment increased protein fermentation.

### Gene Expression in the Ileal Tissue

**FIGURE 7:**
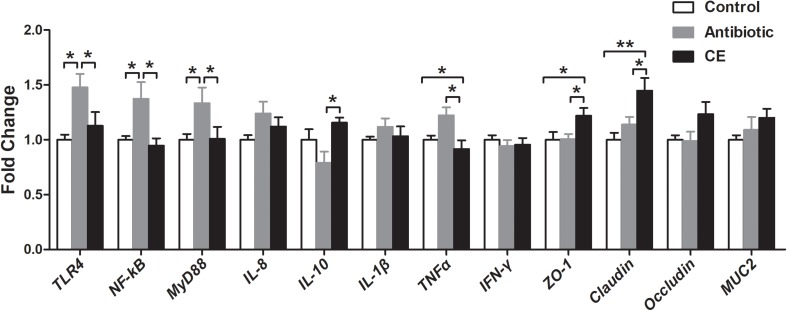
Effect of the dietary supplementation with citrus extract on the relative mRNA expression of genes related to TLR signaling pathway, cytokines, and barrier function in the ileal tissue of Chinese yellow-feathered broilers. Asterisks indicate statistical differences between different groups (one-way ANOVA with a Tukey *post hoc* test): ^∗^*P* < 0.05, ^∗∗^*P* < 0.01. CE, citrus extract; TLR, toll-like receptor.

The alteration of the gut microbial community and their metabolites could regulate intestinal epithelial gene expression. Thus, the mRNA expressions of genes involved in toll-like receptor (TLR), cytokines production, and mucosal defense were analyzed in the ileum. As shown in Figure 7, antibiotic treatment significantly up-regulated the relative mRNA expression of *TLR4* and its downstream signal response genes (*MyD88* and *NF-*κ*B*) compared with those in the control and CE groups (*P* < 0.05). Dietary supplementation with CE down-regulated the relative mRNA expression of *TNF*α compared with that in the antibiotic group (*P* < 0.05), whereas it up-regulated *IL-10*, *ZO-1*, and *Claudin* expression (*P* < 0.05). Moreover, CE treatment also up-regulated *ZO-1* and *Claudin* expression compared with that in the control group (*P* < 0.05). There was no significant difference in the expression of *IL-8*, *IL-1*β, *IFN-*γ, *occludin*, and *MUC2* among the three groups (*P* > 0.05).

### Correlation Analysis Between Mucosal Gene Expression With Ileal Microbes or Their Metabolites

**FIGURE 8:**
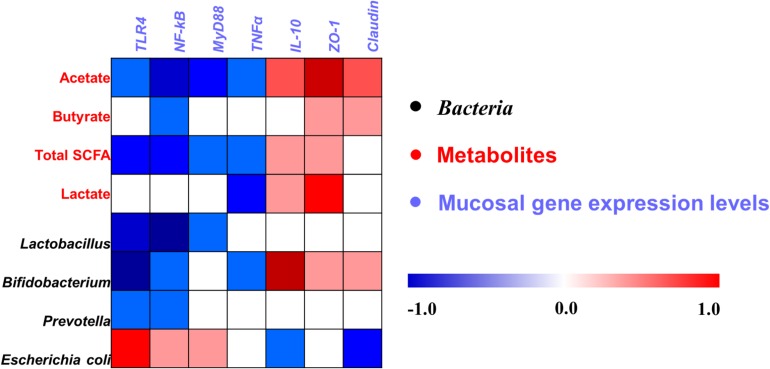
Pearson’s correlation analysis between the abundance of ileal microbiota (qPCR), microbial metabolites, and mucosa gene expression level affected by dietary treatment. Cells are colored based on the correlation coefficient between the significantly changed bacteria (the numbers of bacteria), metabolites (concentrations), and mucosal gene expression level. The intensity of the colors represents the degree of association. Red represents a significant positive correlation (*P* < 0.05), blue represents significantly negative correlation (*P* < 0.05), and white shows that the correlation was not significant (*P* > 0.05). Total SCFA, total short-chain fatty acid.

A Pearson correlation analysis was carried out to determine whether there was any relationship among mucosal gene expression and main microbial numbers and the concentrations of metabolites (Figure 8). Correlation analysis revealed that the mRNA expression level of *TLR-4* and *NF-*κ*B* were positively correlated with the number of *E. coli* (*P* < 0.05), whereas it negatively correlated with the concentrations of acetate and total SCFAs, and the number of *Lactobacillus*, *Bifidobacterium*, and *Prevotella* (*P* < 0.05). The *MyD88 mRNA* expression level was positively correlated with the number of *E. coli* (*P* < 0.05), whereas it negatively correlated with the concentrations of acetate and total SCFAs, and the number of *Lactobacillus* (*P* < 0.05). The mRNA expression level of *TNF*α was negatively correlated with the concentrations of acetate, total SCFAs, and lactate, and the number of *Bifidobacterium* (*P* < 0.05). The mRNA expression level of *IL-10* and *ZO-1* was positively correlated with the concentrations of acetate, total SCFAs, and lactate, and the number of *Bifidobacterium* (*P* < 0.05), and the *ZO-1* expression level also positively correlated with the butyrate concentration (*P* < 0.05), whereas the *IL-10* expression level negatively correlated with the number of *E. coli* (*P* < 0.05). Meanwhile, the *Claudin* mRNA expression level was positively correlated with the acetate and butyrate concentrations (*P* < 0.05), whereas it negatively correlated with the number of *E. coli* (*P* < 0.05). Additionally, the correlation between microbes and the concentrations of metabolites in the cecum was also analyzed and is shown in Supplementary Figure S4. Overall, these results indicated that the alteration in the ileal digesta microbiota and metabolites was correlated with the changes of epithelial gene expression in broilers.

## Discussion

The present study employed a whole growth stage continuous feed strategy to evaluate the effects of dietary supplementation with CE on the microbial community, microbial metabolite profiles, and expression of immune-related genes in the intestine of yellow-feathered broilers. The results showed that dietary supplementation with CE dramatically increased the number of *Bifidobacterium* and up-regulated the mRNA expression of intestinal barrier genes (*ZO-1* and *Claudin*) in the ileum, whereas it decreased the number of *E. coli*. Meanwhile, CE supplementation increased the number of *Lactobacillus* and the concentrations of lactate and SCFAs in the cecum, whereas it decreased the concentrations of protein fermentation products (ammonia, *p*-cresol, indole, total amines, spermidine, methylamine, and tyramine). These findings further highlight the key role of CE in regulating intestinal microbiota, metabolic profiles, and mucosal immune system, suggesting that CE may act as an efficient alternative of antibiotics for yellow-feathered broiler production.

### Citrus Extract Altered the Intestinal Microbiota of Yellow-Feathered Broilers

The gastrointestinal microbes of mammals play an important role in prevention of infectious diseases, regulation of nutrient digestion and metabolism, maintenance of intestinal morphology, and immune homeostasis of the host (Nicholson et al., 2005; Hooper et al., 2012; Song et al., 2019b). Applying functional substances to the diet of animals is an advantageous strategy for the modulation gastrointestinal microbiota and maintenance of host health. Citrus products have antimicrobial activity against *E coli* and *S. typhimurium* in poultry production and have a positive effect on intestinal health (Nannapaneni et al., 2008; O’Bryan et al., 2008). In the current study, we found that dietary supplementation with CE selectively regulated intestinal microbiota, including stimulating bacterial species with beneficial function (*Bifidobacterium* and *Lactobacillus*) and inhibiting the number of potential pathogen (*E. coli*). *Bifidobacterium* is recognized as a beneficial bacterium and potential probiotic, and a number of species can produce acetate and lactate (Gibson et al., 2004) and have a positive effect on intestinal health of both humans and animals and offer the ability to normalize the ratio of pro-inflammatory and anti-inflammatory cytokines (O’Mahony et al., 2005). *Lactobacillus* is well known as a potentially beneficial species in the intestine, which can inhibit the colonization of potential pathogenic groups by competing with the epithelial binding sites and nutrients, and the productions of antimicrobial factors including lactate and bacteriocins, thus maintaining the homeostasis of the intestinal environment of host (Yu et al., 2018). *E. coli* is an opportunistic pathogenic bacteria that has been shown to be positively associated with numerous infections and the development of diseases, including bacillary dysentery or colitis disease (Barnich et al., 2007). Thus, these results indicate that dietary inclusion of CE may be beneficial for the intestinal health for broilers.

To date, the research for the effect of CE on intestinal microbiota is still limited. The beneficial functions of plant extract mainly depended on their specific bioactive components (such as organic acids, polysaccharide, and flavone), which can synthesize as antimicrobial agents against microbial infection and alter their composition (Lillehoj et al., 2018). In our study, CE contained plentiful contents of total flavone (about 2.48%), which can target and modulate microbiota composition. Dietary flavonoid intake significantly increased the abundance of *Bifidobacterium* in humans (Klinder et al., 2016) or mice (Espley et al., 2013). CE also has a high concentration of polysaccharide (about 1.2% in the current study), which has the capability to change the composition and diversity of intestinal microbiota, such as increase the abundance of *Lactobacillus* in mice (Li et al., 2019). Additionally, CE is also a potential source of organic acids (including 1.3% citric acid and 0.68% chlorogenic acid), which can decrease the pH in the gastrointestinal tract and then inhibit the growth of some pathogenic bacteria (such as *E. coli*) owing to their susceptibility to low pH (Feng et al., 2018; Zhang Y. et al., 2018). Thus, the intestinal microbiota alteration of broilers in response to administering CE may be attributed to the flavonoid, polysaccharide, and/or organic acid contents, whereas the underlying mechanisms should be further studied.

### Citrus Extract Increased Microbial Fermentation of Carbohydrate but Decreased Fermentation of Protein

The microbial metabolite profiles in the intestinal digesta can reflect the microbial activity and intestinal health. SCFAs and lactate are primarily fermentation products of the carbohydrate metabolism by bacteria in the gut. In the current study, CE significantly increased the concentrations of lactate, total SCFA, acetate, and butyrate in the cecum, which suggests that CE increased the carbohydrate fermentation by bacteria. *Bifidobacterium* and *Lactobacillus* are the main acetate, butyrate, and lactate producers in the gut (Gao et al., 2018). Increase in SCFAs and lactate concentrations was accompanied by an increase of the number of *Bifidobacterium* and *Lactobacillus*. Thus, one potential explanation for increased cecal digesta SCFAs and lactate concentrations may be due to increase the abundance or numbers of SCFA- and lactate-producing bacteria. SCFAs and lactate can exert many beneficial effects for host health. Acetate can be used as energy substrate of peripheral tissues, butyrate exerts an anti-inflammatory function and is the main energy source of colonic epithelial cells (Yu et al., 2018), and lactate can reduce the pH value in the gastrointestinal tract and inhibit the multiplication of pathogens that invade the gut (such as *E. coli*) (Gao et al., 2018; Yu et al., 2018). Thus, the increase of total SCFAs, acetate, butyrate, and lactate concentrations in the current study suggests a host-friendly gut environment after CE treatment.

Nitrogenous compounds, such as undigested protein and amino acids, can also be fermented by intestinal bacteria and form putrefactive catabolites, such as ammonia, biogenic amines, phenol, and indole compounds. In the current study, the decrease in cecal ammonia, biogenic amines, phenol, and indole levels suggests that bacterial deamination and decarboxylation of amino acids were affected after CE administration. A high ammonia level has been shown to exert a negative effect on the health of the hosts, such as inhibiting the growth and differentiation of intestinal epithelial cells and increasing the incidence of diarrhea in the host (Dong et al., 1996). Additionally, high concentrations of biogenic amines (tyramine), indole, and *p*-cresol also exert adverse impacts on gut health and are regarded as co-carcinogens and colon cancer promoters (Nowak and Libudzisz, 2006; Davila et al., 2013). Thus, the decrease in the concentrations of ammonia, biogenic amines, indole, and phenolic compounds via CE supplementation may have a beneficial effect on gut health. Overall, together with the increase in lactate and SCFAs, these findings indicate that CE changed the microbial metabolic activity, increased microbial fermentation of carbohydrate, and decreased microbial protein catabolism.

### Citrus Extract Affected the Ileal Mucosal Response Involved in the Intestinal Barrier Function

The intestinal barrier performs the essential function of defense against the passage of pathogenic agents and luminal antigens into the gastrointestinal epithelium while enabling the acquisition of dietary nutrients (Broom, 2018). In the present study, CE supplementation up-regulated the mRNA expression of intestinal barrier genes in the ileum compared with that in control and antibiotic groups, such as *ZO-1* and *Claudin*. This suggests that CE may improve the integrity of the intestinal epithelium, consequently generating a host-friendly gut environment, which could help defend against pathogen infection. The changes in intestinal microbes and associated metabolites can both positively and negatively affect the histological function of the gastrointestinal epithelium, such as intestinal barrier permeability. Previous studies have shown that a number of pathogens, such as the *Enterotoxigenic E. coli* K88, can impair intestinal barrier functions by down-regulating *ZO-1* and *occludin* gene and protein expression (Wang et al., 2017), whereas *Lactobacillus reuteri* LR1 up-regulated the expression of *ZO-1* and *occludin* gene in piglets (Yi et al., 2018). Moreover, some bacterial fermentation product, such as butyrate, can maintain gut integrity. As described above, dietary inclusion of CE increased the numbers of *Bifidobacterium* and *Lactobacillus* and the concentrations of total SCFAs, acetate, and butyrate and decreased the number of *E. coli*. Our previous study also revealed that the *Bifidobacterium* and *Lactobacillus* numbers and butyrate concentration were positively correlated with mRNA levels of *ZO-1* and *occludin* (Yu et al., 2019). Therefore, we might speculate that the high number of *Bifidobacterium* and *Lactobacillus*, SCFAs, and butyrate concentrations in the CE group may be the factors accounting to the up-regulation of intestinal barrier gene expression, although the underlying mechanism required further clarification.

### Antibiotic Treatment Induced Intestinal Microbiota Dysbiosis, Altered Fermentation Profiles of Microbial Metabolism, and Disturbed the Innate Immune Homeostasis

Antibiotics can cause gut microbiota dysbiosis, inhibit the innate immune defenses, and lead to increased pathogen colonization and disease susceptibility (Zhang C.J. et al., 2018). In the current study, antibiotic treatment increased the abundance and the number of potential pathogens, while decreasing those with beneficial function, which was consistent with previous studies (Yu et al., 2017a, 2018). Among the affected bacterial groups, many bacterial species have previously been related to increased disease risk in humans or animals. For example, antibiotic treatment decreased the abundances of *Barnesiella* and *Blautia*, and the numbers of *Lactobacillus*, *Ruminococcus*, *Prevotella*, and *Bifidobacterium*, and increased the abundance of *Alistipes* and the number of *E. coli*. *Barnesiella* and *Blautia* are SCFA producers (Zhang et al., 2012) and have the ability to protect the integrity of the intestinal barrier function and alleviate dextran sulfate sodium-induced inflammation (Lu et al., 2018). *Ruminococcus* and *Prevotella* are key bacterial groups for the degradation of dietary fibers and polysaccharide to regulate the host metabolism (Wang et al., 2018). *Alistipes* can directly elevate inflammation levels and induce mucosal injuries in goats (Ye et al., 2016). The potentially beneficial function of *Lactobacillus* and *Bifidobacterium* and the potential adverse function of *E. coli* have been mentioned above. Taken together, these results point to a potentially detrimental impact of antibiotic treatment on the composition of intestinal microbiota in broilers.

Corresponding to the alteration of intestinal microbiota, antibiotics showed a marked impact on the microbial metabolism of carbohydrate and amino acids in the cecum, as indicated by the decrease in the concentrations of lactate and most SCFAs, and the increase in the concentrations of BCFA, most amines, and *p*-cresol. This is consistent with previous studies (Gao et al., 2018; Pi et al., 2018; Yu et al., 2018), which observed that antibiotic treatment led to a decrease in most SCFA concentrations, whereas it increased amine concentrations in pigs. Overall, the findings of this study provide clear evidence for a shift of the microbial metabolic activity, with lower microbial carbohydrate fermentation and higher microbial catabolism of amino acids after antibiotic treatment.

The alteration of intestinal microbes and their metabolites could regulate the immune homeostasis of intestinal mucosa. In the present study, we found that antibiotic treatment up-regulated the mRNA expression of *TLR4*, *NF-*κ*B*, *MyD88*, and pro-inflammatory cytokines *TNF*α. TLR4 can transfer signals to NF-κB via MyD88-dependent pathway and then induce the activation of pro-inflammatory cytokines (Zhang et al., 2017). Thus, the increased gene expression levels of pro-inflammatory cytokines after antibiotic treatment may be attributed to an up-regulation of the TLR4-MyD88-NF-κB signaling pathway of broilers. Collectively, together with the alteration of intestinal microbiota, fermentation profiles of microbial metabolism, and the mucosal gene expression levels, our results indicate that antibiotic treatment induced the gut microbiota dysbiosis, altered the microbial metabolism, and inhibited the innate immune defenses, likely toward a host-adverse gut environment.

## Conclusion

In conclusion, this study demonstrated that dietary supplementation with CE changed the intestinal microbial composition, microbial metabolite profiles, and immune status of broilers, likely toward a host-friendly gut environment. Intestinal microbes, such as the numbers of *Bifidobacterium* and *Lactobacillus*, increased after CE supplementation, whereas the number of *E. coli* decreased. Meanwhile, CE markedly increased the concentrations of lactate and SCFAs, whereas it decreased the concentrations of amino acid fermentation products. The expression of intestinal barrier genes (*ZO-1* and *Claudin*) was increased. However, antibiotic treatment induced gut microbiota dysbiosis, altered the microbial metabolism, and disturbed the innate immune homeostasis, likely toward an unhealthy gut environment. These findings suggest that CE may act as an efficient antibiotic alternative for yellow-feathered broiler production.

## Data Availability Statement

The datasets generated for this study can be found in the following repository. The raw reads in this study were submitted to the National Center of Biotechnology Information (NCBI) Sequence Read Archive (SRA) database under accession numbers SRR9074899–SRR9074916.

## Ethics Statement

The experimental proposals and procedures for the care and treatment of the broilers were approved by the Animal Care and Use Committee of the Guangdong Academy of Agricultural Sciences (authorization number GAASIAS-2016-017).

## Author Contributions

MY, WC, and XM conceived and designed the whole trial. ZL and YC conducted the broiler trials. MY and ZL conducted laboratory analyses. MY, XM, and GW wrote the manuscript.

## Conflict of Interest

The authors declare that the research was conducted in the absence of any commercial or financial relationships that could be construed as a potential conflict of interest.

## Footnotes

1 http://drive5.com/uparse/
