# Supplementary material for: Dietary Supplementation With Citrus Extract Altered the Intestinal Microbiota and Microbial Metabolite Profiles and Enhanced the Mucosal Immune Homeostasis in Yellow-Feathered Broilers
Source: Front Microbiol. 2019 Nov 26;10:2662. doi: 10.3389/fmicb.2019.02662 (PMC6887900; doi:10.3389/fmicb.2019.02662)
Supplement: Supplementary file 1 [file Table_1.docx]

**Dietary supplementation with citrus extract altered the intestinal microbiota, microbial metabolite profiles, and enhanced the mucosal immune homeostasis in yellow-feathered broilers**

Miao Yu, Zhenming Li, Weidong Chen, Gang Wang, Yiyan Cui, Xianyong Ma^†^

Institute of Animal Science, Guangdong Academy of Agricultural Sciences; State Key Laboratory of Livestock and Poultry Breeding; Key Laboratory of Animal Nutrition and Feed Science in South China, Ministry of Agriculture; Guangdong Public Laboratory of Animal Breeding and Nutrition; Guangdong Engineering Technology Research Center of animal Meat quality and Safety Control and Evaluation, Guangzhou 510640, Guangdong, People’s Republic of China

Corresponding authors: Xianyong Ma

Address: No.1, Dafeng First St., Wushan, Tianhe district, Guangzhou, Guangdong,

China.

E-mail: [maxianyong@gdaas.cn](mailto:maxianyong@gdaas.cn)

**Supplementary material**

**Summary**

The supporting information includes 2 supplementary table and 3 supplementary figures.

**Table S1** Primers used for quantification in this study

| Target | Primer sequence 5′-3′ | Reference |
| --- | --- | --- |
| Total bacteria | Forward: CGGTGAATACGTTCYCGG  Reverse: GGWTACCTTGTTACGACTT | Suzuki, et al., 2000 |
| Firmicutes | Forward: GGAGYATGTGGTTTAATTCGAAGCA | Guo, et al., 2008 |
|  | Reverse: AGCTGACGACAACCATGCAC |  |
| Bacteroidetes | Forward: GGARCATGTGGTTTAATTCGATGAT | Guo, et al., 2008 |
|  | Reverse: AGCTGACGACAACCATGCAG |  |
| *Clostridium* cluster IV | Forward: GCACAAGCAGTGGAGT | Matsuki, et al., 2004 |
|  | Reverse: CTTCCTCCGTTTTGTCAA |  |
| *Clostridium* cluster XIVa | Forward: CGGTACCTGACTAAGAAGC | Bartosch, et al., 2004 |
|  | Reverse: AGTTTYATTCTTGCGAACG |  |
| *Escherichia.coli* | Forward: CATGCCGCGTGTATGAAGAA | Huijsdens, et al., 2002 |
|  | Reverse: CGGGTAACGTCAATGAGCAAA |  |
| *Bifidobacterium* | Forward: TCGCGTCYGGTGTGAAAG | Walker, et al., 2011 |
|  | Reverse: GGTGTTCTTCCCGATATCTAC |  |
| *Lactobacillus* | Forward: AGCAGTAGGGAATCTTCCA | Khafipour, et al., 2009 |
|  | Reverse: ATTCCACCGCTACACATG |  |
| *Bacteroides-Prevotella* | Forward: GAGAGGAAGGTCCCCCAC | Layton, et al., 2006 |
|  | Reverse: CGCTACTTGGCTGGTTCAG |  |
| *Ruminococcus* | Forward: GAAAGCGTGGGGAGCAAACAGG | Verma, et al., 2010 |
|  | Reverse: GACGACAACCATGCACCACCTG |  |

**Table S2.** Primers used for host genes in this study

| Target | Primer sequence 5′-3′ | Reference |
| --- | --- | --- |
| *β-actin* | Forward: ATCCGGACCCTCCATTGTC | Ma, et al., 2018 |
|  | Reverse: AGCCATGCCAATCTCGTCTT |  |
| *IL-1β* | Forward: ACTGGGCATCAAGGGCTACA | Ma, et al., 2018 |
|  | Reverse: GCTGTCCAGGCGGTAGAAGA |  |
| *IL-8* | Forward: GGCTTGCTAGGGGAAATGA | Wang, et al., 2017 |
|  | Reverse: AGCTGACTCTGACTAGGAAACTGT |  |
| *IL-10* | Forward: ATGAACTTAACATCCAACTGCTC | Xiao, et al., 2018 |
|  | Reverse: TGTTGCCCAGGTCGCCCAT |  |
| *TLR4* | Forward: GTTCCTGCTGAAATCCCAAA | Zhang, et al., 2017 |
|  | Reverse: TATGGATGTGGCACCTTGAA |  |
| *TNF-α* | Forward: TACTCAGGACAGCCTATGCCAACAA | Ma, et al., 2018 |
|  | Reverse: GGAAGGGCAACTCATCTGAACTGG |  |
| *IFN-γ* | Forward: GACAAGTCAAAGCCGCACAT | Ma, et al., 2018 |
|  | Reverse: CAAGTCGTTCATCGGGAGC |  |
| *NF-kB* | Forward: TTGCTGCTGGAGTTGATGTC | Scott, et al., 2016 |
|  | Reverse: TGCTATGTGAAGAGGCGTTG |  |
| *MyD88* | Forward: TGATGCCTTCATCTGCTACTG | Li, et al., 2010 |
|  | Reverse: TCCCTCCGACACCTCTTTCTA |  |
| *Mucin-2* | Forward: TTCATGATGCCTGCTCTTGTG | Guo et al., 2018 |
|  | Reverse: CCTGAGCCTTGGTACATTCTTGT |  |
| *Occludin* | Forward: ACGGCAGCACCTACCTCAA | Guo et al., 2018 |
|  | Reverse: GGGCGAAGAAGCAGATGAG |  |
| *ZO-1* | Forward: CTTCAGGTGTTTCTCTTCCTCCTC | Guo et al., 2018 |
|  | Reverse: CTGTGGTTTCATGGCTGGATC |  |
| *Claudin-1* | Forward: CATACTCCTGGGTCTGGTTGGT | Guo et al., 2018 |
|  | Reverse: GACAGCCATCCGCATCTTCT |  |

**Supplemental Figure S1** Rarefaction curves comparing the number of sequences with the number of phylotypes found in the 16S rRNA gene libraries from the microbiota in the digesta of the cecum of Chinese yellow-feathered broilers. Abbreviations: CE, citrus extract.


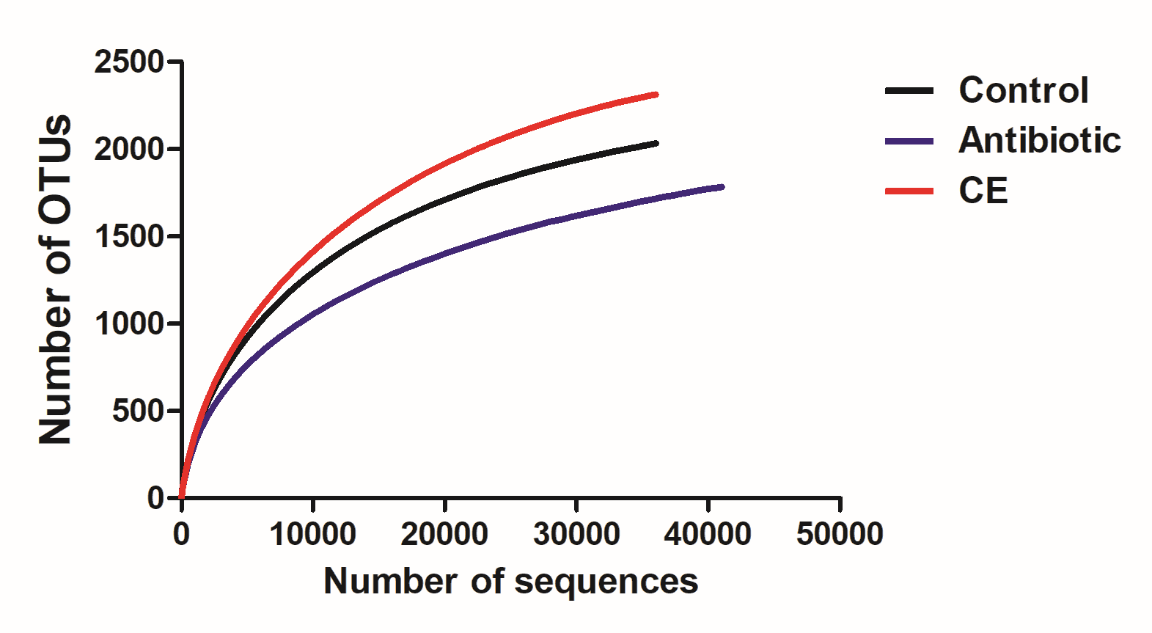


**Supplemental Figure S2** Influence of citrus extract on the diversity of cecal bacterial community at the 3% dissimilarity level. The values are expressed as the means ± SEM, with six yellow-feathered broilers per group. Abbreviations: CE, citrus extract.


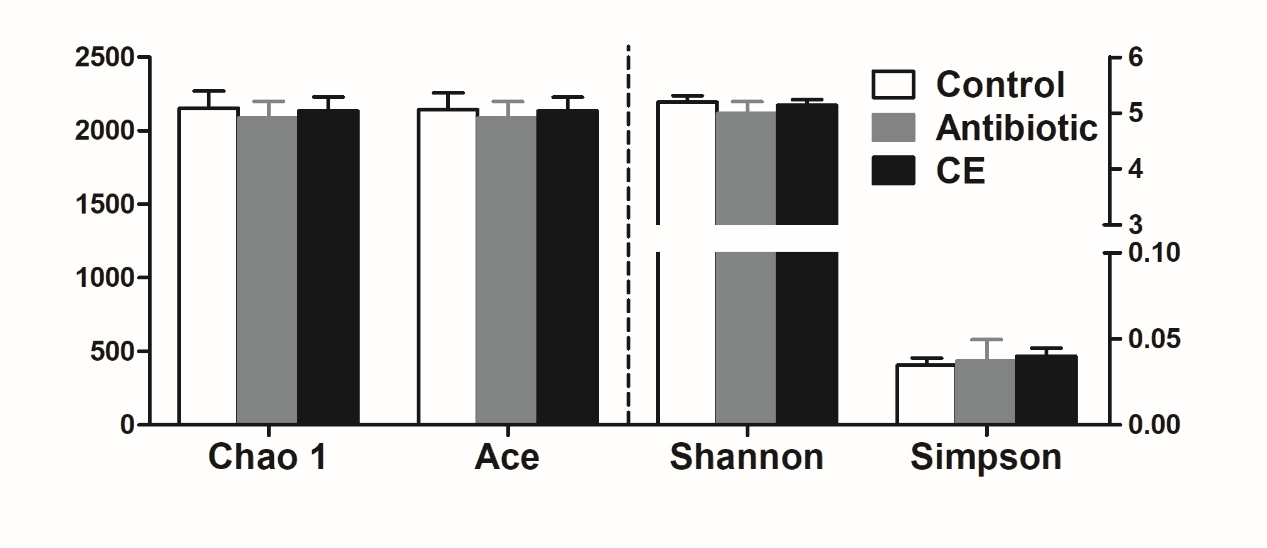


**Supplemental Figure S****3** Influence of citrus extract on the 30 most abundant genera in the cecal digesta of Chinese yellow-feathered broilers. The color represents the relative abundance of bacteria. Abbreviations: CE, citrus extract.


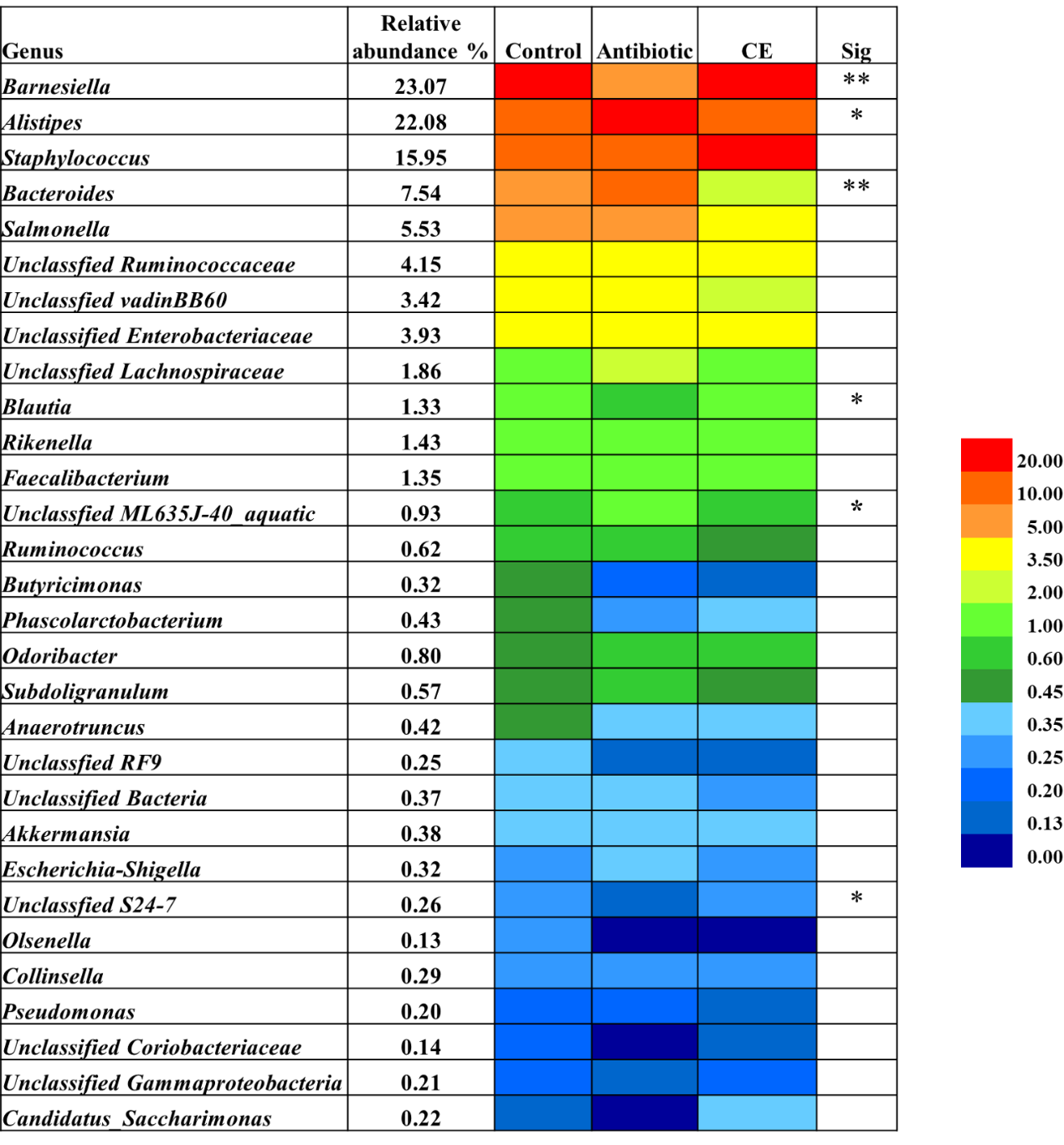


**Supplemental Figure S4** Spearman’s correlation analysis between the abundance of cecal microbiota (at the genus level and qPCR) and microbial metabolites affected by dietary treatment. Cells are colored based on the correlation coefficient between the significantly changed bacteria (the relative abundance and the numbers of bacteria) and metabolites (concentrations). The intensity of the colors represents the degree of association. Red represented a significant positive correlation (*P* < 0.05), blue represents significantly negative correlation (*P* < 0.05), and white shows that the correlation was not significant (*P* > 0.05). Total SCFA: total short-chain fatty acids.


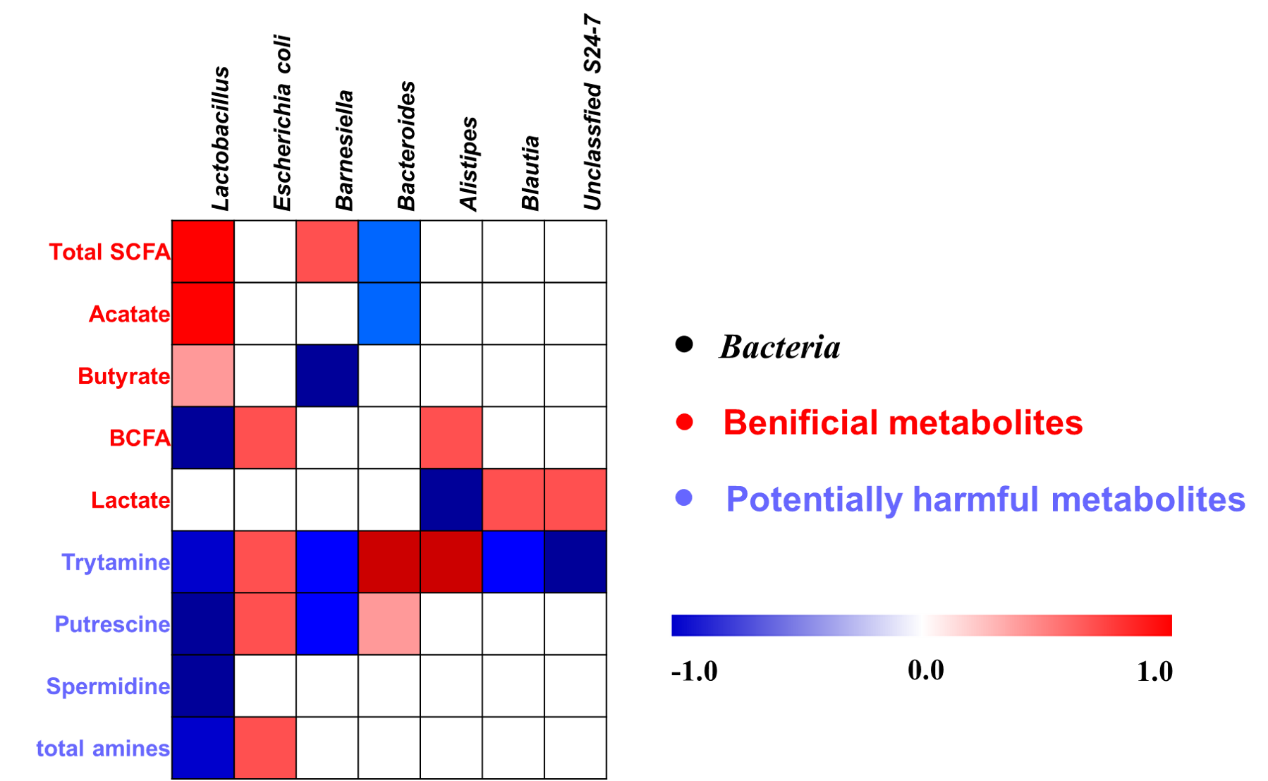


**References**

Bartosch, S., A. Fite, G. T. Macfarlane, and M. E. McMurdo. (2004) Characterization of bacterial communities in feces from healthy elderly volunteers and hospitalized elderly patients by using real-time PCR and effects of antibiotic treatment on the fecal microbiota, *Appl. Environm. Microbiol.* 70:3575-3581.

Guo, S., Q. Cheng, Y. Li, R. Duan, Y. Hou, D. Y, and B. Ding. (2018) Effects of dietary coated-oleum cinnamomi supplementation on the immunity and intestinal integrity of broiler chickens, *Anim. Sci. J.* 89:1581–1590.

Guo, X., X. Xia, R. Tang, J. Zhou, H. Zhao, and K. Wang, (2008) Development of a real-time PCR method for Firmicutes and Bacteroidetes in faeces and its application to quantify intestinal population of obese and lean pigs, *Letters. Appl. Microbiol.* 47:367-373.

Huijsdens, X. W., R. K. Linskens, M. Mak, S. G. Meuwissen, C. M. Vandenbroucke-Grauls, and P. H. Savelkoul, (2002) Quantification of bacteria adherent to gastrointestinal mucosa by real-time PCR, *J. Clin. Microbiol*. 40:4423-4427.

Khafipour, E., S. Li, J. C. Plaizier, and D. O. Krause, (2009) Rumen microbiome composition determined using two nutritional models of subacute ruminal acidosis, *Appl. Environm. Microbiol.* 75:7115-7124.

Layton, A., L. Mckay, W. Dan, V. Garrett, R. Gentry, and G. Sayler, (2006) Development of bacteroides 16S rRNA gene TaqMan-Based real-time PCR assays for estimation of total, human, and bovine fecal pollution in water, *Appl. Environm. Microbiol.* 72:4214-4224.

Li, P., P. Xia, J. Wen, M. Zheng, J, Chen, J. Zhao, R. Jiang, R. Liu, and G. Zhao, (2010) Up-regulation of the MyD88-dependent pathway of TLR signaling in spleen and caecum of young chickens infected with *Salmonella serovar Pullorum*. *Vet. Microbiol.* 143:346-351.

Ma, Y., W. Wang, H. Zhang, J. Wang, W. Zhang, Gao, J., S. Wu, and G. Qi. (2018) Supplemental *Bacillus subtilis* DSM 32315 manipulates intestinal structure and microbial composition in broiler chickens, *Sci. Rep*. 8:15358.

Matsuki, T., K. Watanabe, J. Fujimoto, T. Takada, and R. Tanaka, (2004) Use of 16S rRNA gene-targeted group-specific primers for real-time PCR analysis of predominant bacteria in human feces, *Appl. Environm. Microbiol.* 70:7220-7228.

Scott, A., K. P. Vadalasetty, E. Sawosz, M. Łukasiewicz, R. K. P. Vadalasetty, S. Jaworski, and A. Chwalibog, (2016) Effect of copper nanoparticles and copper sulphate on metabolic rate and development of broiler embryos. *Anim. Feed. Sci. Tech*. 10:151-158.

Suzuki, M. T., L. T. Taylor, and E. F. Delong. (2000) Quantitative analysis of small-subunit rRNA genes in mixed microbial populations via 5′-nuclease assays. *Appl. Environ. Microbiol.* 66:4605-4614.

Verma, R., A. K. Verma, V. Ahuja, and J. Paul, (2010) Real-time analysis of mucosal flora in patients with inflammatory bowel disease in India, *J. Clin. Microbiol.* 48:4279-4282.

Walker, A. W., J. Ince, S. H. Duncan, L. M. Webster, G. Holtrop, X. Ze, D. Brown, M. D. Stares, and A. Bergerat, (2011) Dominant and diet-responsive groups of bacteria within the human colonic microbiota, *ISME. J.* 5:220-230.

Wang, H., X. Ni, X. Qing, L. Liu, J. Lai, A. Khalique, G. Li, K. Pan, B. Jing, and D. Zeng. (2017) Probiotic enhanced intestinal immunity in broilers against subclinical necrotic enteritis*. Front. Immunol.* 8:1592.

Xiao, M., Y. Mi, L. Liu, C. Lv, W. Zeng, C. Zhang, and J. Li, (2018) Taurine regulates mucosal barrier function to alleviate lipopolysaccharide-induced duodenal inflammation in chicken. *Amino. Acids.* 50:1637-1646.

Zhang, Q., S. D. Eicher, K. M. Ajuwon, and T. J. Applegate, (2017) Development of a chicken ileal explant culture model for measurement of gut inflammation induced by lipopolysaccharide. *Poultry. Sci.* 9:3096–3103.
